# The Efficacy of Ozonated Olive Oil in Crown Lengthening Procedures for Anterior Teeth: A Case Report

**DOI:** 10.7759/cureus.66480

**Published:** 2024-08-08

**Authors:** Sakshi V Kotecha, Priyanka Jaiswal, Shweta Bhagat

**Affiliations:** 1 Department of Periodontology, Sharad Pawar Dental College, Datta Meghe Institute of Higher Education and Research, Wardha, IND; 2 Department of Dentistry, Sharad Pawar Dental College, Datta Meghe Institute of Higher Education and Research, Wardha, IND

**Keywords:** antibacterial agents, periodontal dressings, ozonated olive oil, mechanical trauma, clinical crown height, periodontium

## Abstract

Ensuring the health of the gums and supporting structures (periodontium) is crucial during dental restoration procedures to achieve optimal function and appearance of the teeth. Understanding the anatomy, the impact of restorative materials, and their interaction with the periodontium is essential for successful treatment outcomes. Crown lengthening is a surgical procedure that involves the removal of gingiva and bone tissue to expose more of the tooth structure, thereby increasing the visible portion of the tooth (clinical crown height). To protect the wound from mechanical trauma and stability of the surgical site during the healing process, periodontal dressing can be applied after surgery. Ozone therapy has demonstrated its effectiveness in promoting the healing of various types of wounds, including chronic and difficult-to-heal wounds, as an antibacterial agent and in modulating the immune system. Thus, this study aimed to compare, evaluate, and assess the use of periodontal dressing with that of ozonated olive oil after the crown lengthening procedure.

## Introduction

Hemostasis, inflammation, proliferation, and remodeling are the four molecular phases of wound healing, and they are all involved in this intricate, multifaceted process [1]. Hemostasis is aided by platelet aggregation and fibrin clot formation [2], with calcium ions (Ca^2+^) being essential for clot stabilization [3]. The inflammatory phase is influenced by the flow of inflammatory cells and substances into the damaged area [4]. In this instance, neutrophils debride the wound while macrophages release cytokines near the location of the lesion. Fibroblasts move into the wound during the proliferative phase, which is a sign of wound healing. They produce a new extracellular matrix to start the process of reepithelialization [2]. Wound contraction and collagen deposition are characteristics of remodeling [5]. Periodontal crown lengthening surgery (CLP) is a procedure in which the wound heals by secondary intention if a simple gingivectomy is performed and the osteotomy is not required. On the other hand, it has been demonstrated that applying a periodontal dressing after gingivectomy and reverse bevel flap surgeries does not significantly enhance the clinical result and may even make postoperative pain more prevalent. Healing similar to that after using a periodontal dressing might be accomplished if plaque collection on the gingivectomy wound area and teeth was prevented [6].

Open wounds are frequently covered with periodontal dressings after periodontal therapy [7]. Today's periodontal dressings are not made of substances that hasten wound healing; instead, they only shield the tissue surrounding the lesion rather than supplying components that promote healing [8]. An essential step in the healing of gum wounds is preserving homeostasis and repairing the integrity of injured tissue. The therapeutic administration of ozone gas in air, water, or oily medium for the treatment of various pathologies is known as ozone therapy. The topical application of ozone therapy has demonstrated efficacy in disinfection and has been used to treat ischemic lesions, fungal, bacterial, and viral infectious disorders, as well as large wounds. It also assists in wound healing and the resolution of inflammation by arresting plaque accumulation after oral surgical procedures [9].

This study aimed to compare, assess, and evaluate the effect of COE-PAK (Alsip, IL: GC America Inc.) periodontal dressing with that of ozonated olive oil in terms of wound healing, tissue response, and pain after the crown lengthening procedure.

## Case presentation

**Figure 1:**
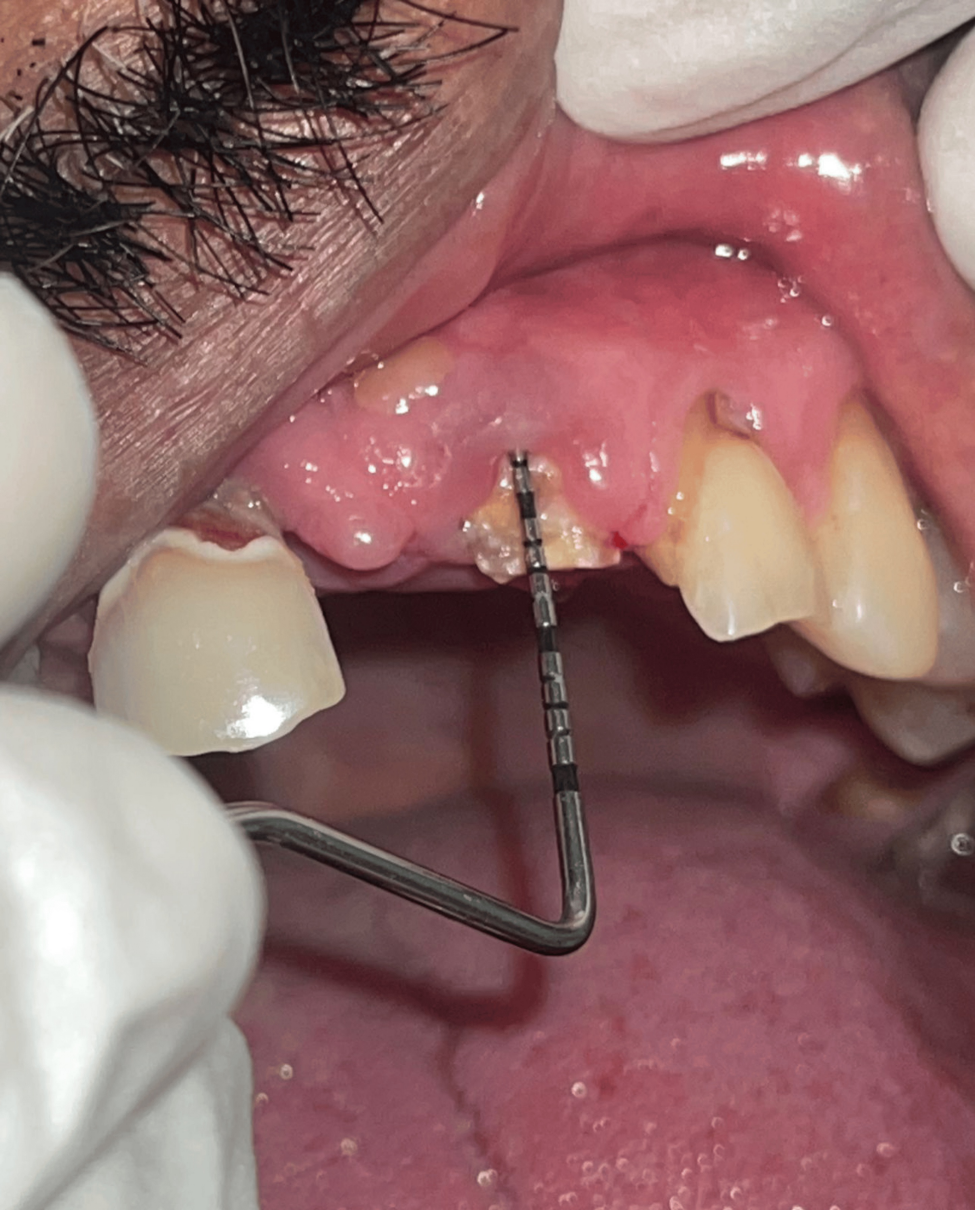
Preoperative photograph of PPD, CAL, and biologic width of the tooth to be treated. PPD: probing pocket depth; CAL: clinical attachment loss

The patient was referred from the Department of Prosthodontics to the Department of Periodontics and Implantology for an evaluation of tooth 21. After complete evaluation and recording probing pocket depth, clinical attachment loss, biologic width, and radiographic bone present, the treatment plan decided was to surgically lengthen the crown for prosthetic rehabilitation (Figure 1). The treatment plan was explained to the patient and informed consent was taken. Biologic width was found to be 1.5 mm.

**Figure 2:**
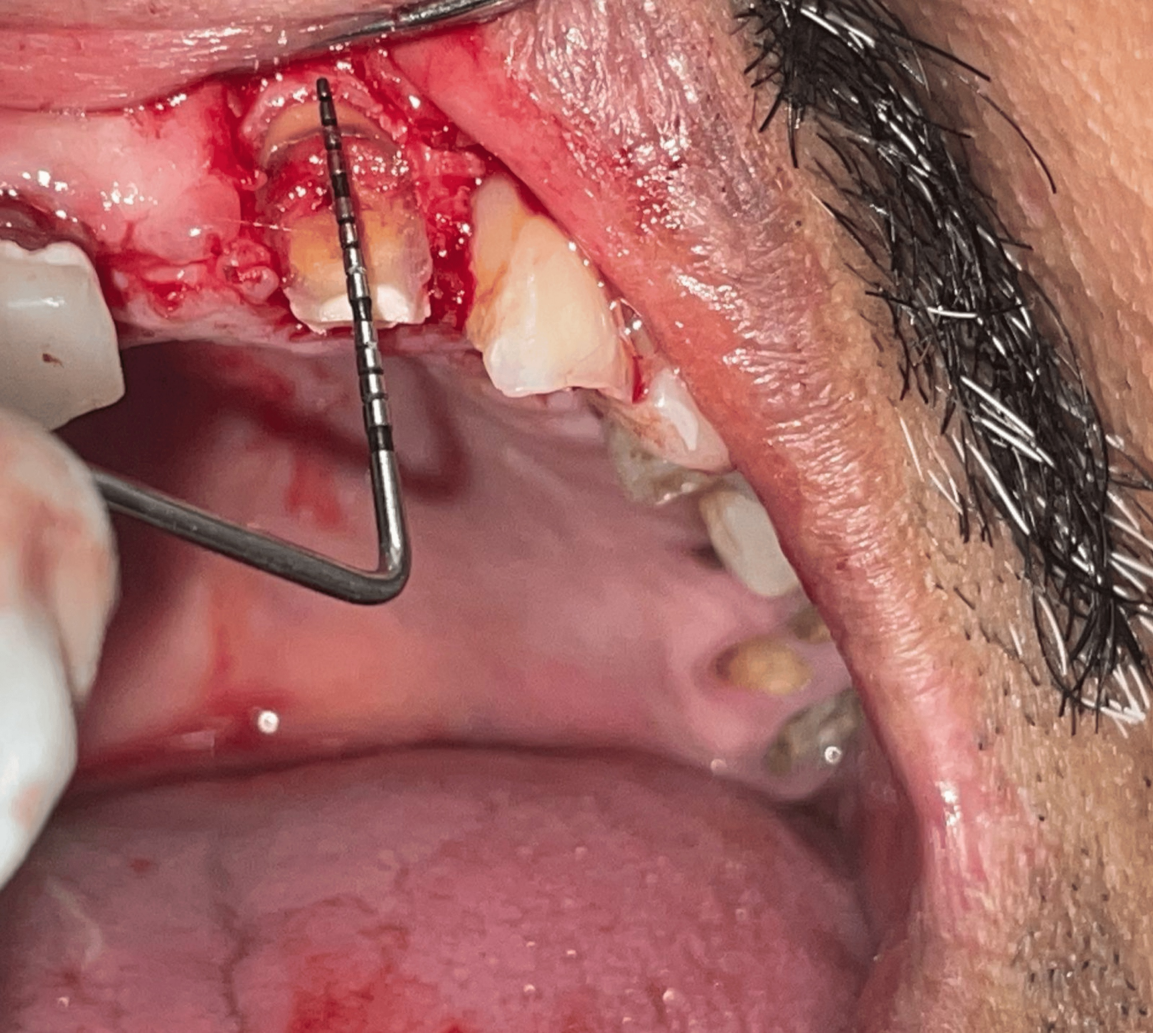
The optimum crown height acquired for retention of crown prosthesis after performing incision and flap reflection.

The crown lengthening procedure was performed under local anesthesia (2% lignocaine hydrochloride) using scalpel and blade. The crevicular incision was extended distally and mesially on tooth 22 to cover half of the edentulous space. An internal bevel incision was then made at the site of tooth 21. A full-thickness mucoperiosteal flap was reflected. The length from bone crest to crown height was measured which was found to be 6 mm and it determined how much tissue was needed to be removed to achieve the desired crown length. The crown lengthening procedure was performed using micromotor handpiece and burs which involved removing a precise amount of bone and soft tissue to expose desired tooth structure (Figure 2).

**Figure 3:**
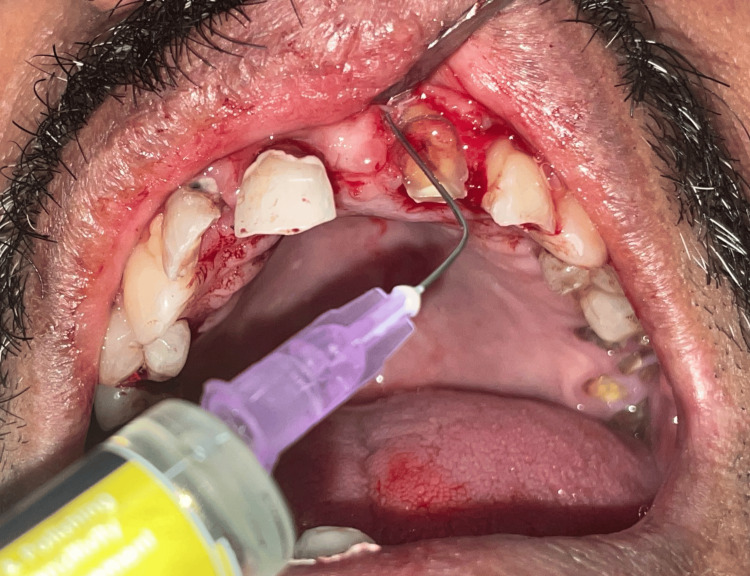
Application of ozonated olive oil on dry tooth surface.

After the surgical procedure, ozone oil (70 mg/mL) (ozone insufflated in olive oil) was applied immediately by the clinician and was also given to the patient to apply as directed on the operated site twice daily for the next seven days (Figure 3). Apically positioned sutures were placed.

**Figure 4:**
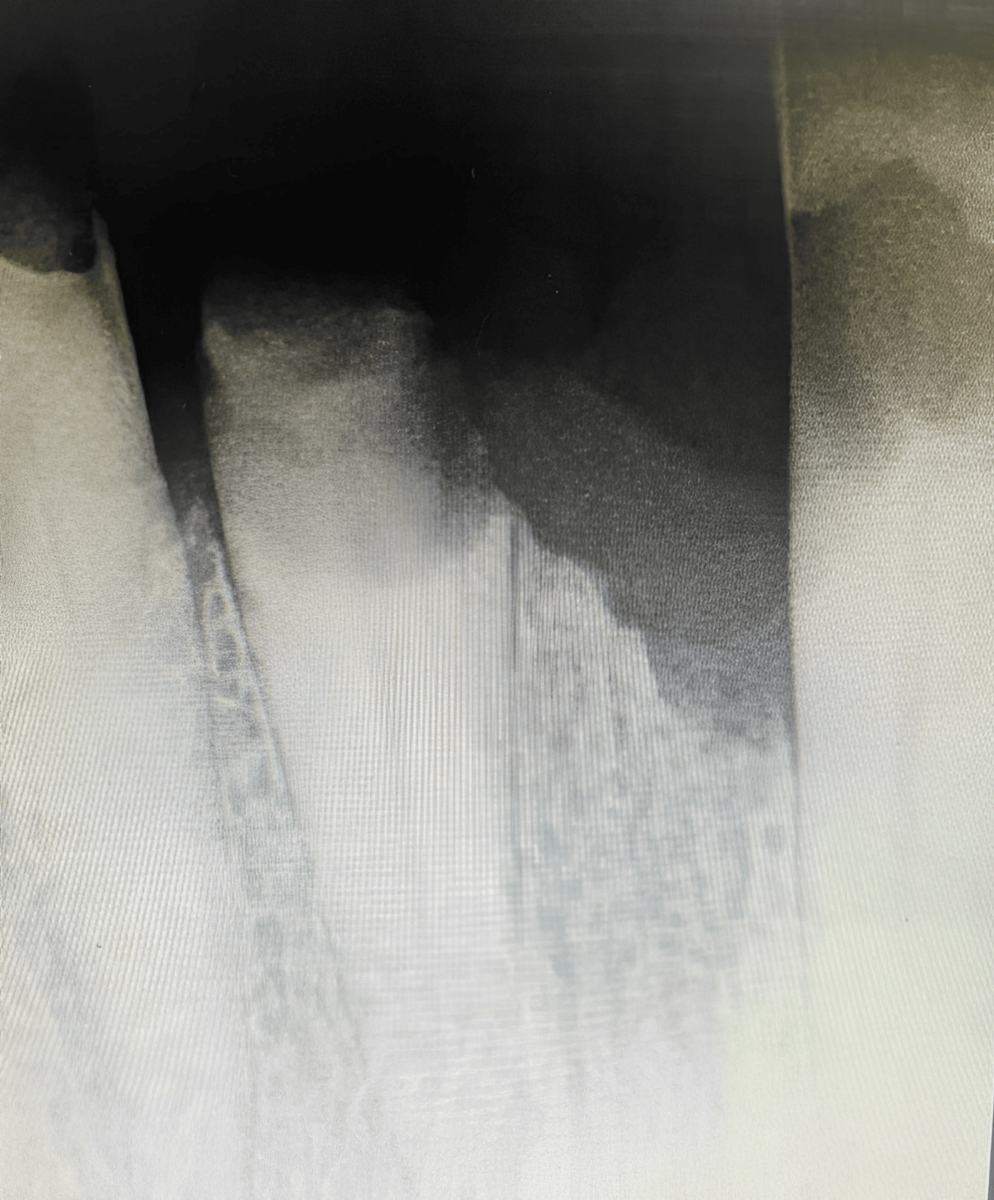
Radiographic view of the treated tooth taken on the seventh day.

Standard postoperative instructions and medications were given to the patient. Patient was recalled after seven days. After one week, tissue response and wound healing (according to the Early Wound Healing Score by Marini et al. in 2018) were evaluated and it was found that the clinical sign of reepithelialization was 3, clinical sign of hemostasis was 2, and clinical sign of inflammation was 2 [10]. Thus, the overall early wound healing score was 7 which inferred better wound healing. The patient was shown the VAS for evaluation of pain and requested to provide a 0-10 pain scale rating. The score given by the patient was 1. At postoperative evaluation, it was found that tissue response was comparably good, wound healing was faster, patient acceptance was better, and pain level was minimal (Figure 4). The clinical crown height obtained was 9 mm.

## Discussion

The foundation for all successful restorative procedures is healthy periodontal tissue. For this purpose, the efficiency of tooth restoration in the future depends on how the periodontal tissues are handled during the restoration process. The use of clinical crown lengthening surgery has two main purposes. The first is a recommendation for lengthening clinical crowns for cosmetic reasons. The second indication, which is also the most prevalent based on actual practice, is the placement of the tooth preparation border gingivally or supragingivally to prevent dental restorations from negatively affecting biological width and causing persistent periodontitis surrounding the applied prosthetic restoration [11]. Crown length, total occlusal convergence degree, and axial surface area are the main variables that affect a dental crown's resistance and retention. Pins inserted into solid tooth structures, boxes, or grooves can provide secondary resistance and retention. A crown placed on a short tooth preparation has a greater tendency to become dislodged compared to a crown on a tooth with the same axial wall height but a lesser diameter. The primary source of retention should be contributed by healthy tooth anatomy [12].

Topical application of ozone oil has proven effectiveness in treating tissue repair due to its antibacterial, immunological, antioxidant, and pain-relieving attributes, along with facilitating wound healing [13]. When human blood comes into contact with ozone, it generates additional interferons (α, β, and γ), interleukins (IL-1, IL-2, IL-6, and IL-8), and tumor necrosis factor (α), these are all essential for the process of epithelization [14]. In the initial postoperative phase, it establishes a barrier covering the surgical site, avoiding infection of the wound. In the end, it lessens pain by covering the exposed nerve terminals as well. A similar study by Patil et al. in 2022 compared effect of ozone oil and non-eugenol periodontal dressing in terms of tissue response, wound healing, and pain after the surgical crown lengthening procedure (CLP) and concluded that ozone oil showed a better reduction in pain perception and can be used alternatively [15]. Thus, ozone oil showed less inflammation, better patient acceptance, and faster healing after seven days.

Difficulties faced during the procedure included the reflection of gingiva due to midline diastema present. According to Rosenberg et al., 4 mm of tooth exposure is preferred for proper accessibility and visibility of the crown [16]. The prognosis of this case is guarded due to the angular defect present.

## Conclusions

In summary, this study highlights the potential of ozone oil as a therapeutic agent that can facilitate faster wound healing, reduce postoperative pain, and serve as a viable alternative to conventional periodontal dressings. These findings warrant further investigation and clinical evaluation to establish the broader applicability of ozone oil in dental and medical practices.
